# Tuberculosis mortality and the male survival deficit in rural South Africa: An observational community cohort study

**DOI:** 10.1371/journal.pone.0185692

**Published:** 2017-10-10

**Authors:** Georges Reniers, Sylvia Blom, Judith Lieber, Abraham J. Herbst, Clara Calvert, Jacob Bor, Till Barnighausen, Basia Zaba, Zehang R. Li, Samuel J. Clark, Alison D. Grant, Richard Lessells, Jeffrey W. Eaton, Victoria Hosegood

**Affiliations:** 1 Department of Population Health, London School of Hygiene & Tropical Medicine, London, United Kingdom; 2 School of Public Health, University of the Witwatersrand, Johannesburg, South Africa; 3 Charles H. Dyson School of Applied Economics and Management, Cornell University, Ithaca, New York, United States of America; 4 Africa Health Research Institute, School of Nursing and Public Health, University of KwaZulu-Natal, Durban, South Africa; 5 Department of Global Health, Boston University, Boston, Massachusetts, United States of America; 6 Department of Global Health and Population, T. H. Chan School of Public Health, Harvard University, Cambridge, Massachusetts, United States of America; 7 Institute of Public Health, University of Heidelberg, Heidelberg, Germany; 8 Department of Statistics, University of Washington, Seattle, United States of America; 9 Department of Sociology, The Ohio State University, Columbus, Ohio; 10 Department of Clinical Research, London School of Hygiene and Tropical Medicine, London, United Kingdom; 11 Department of Infectious Disease Epidemiology, School of Public Health, Imperial College, London, United Kingdom; 12 Social Statistics and Demography, University of Southampton, Southampton, United Kingdom; Tulane University School of Public Health and Tropical Medicine, UNITED STATES

## Abstract

**Background:**

Women live on average five years longer than men, and the sex difference in longevity is typically lower in populations with high mortality. South Africa—a high mortality population with a large sex disparity—is an exception, but the causes of death that contribute to this difference are not well understood.

**Methods:**

Using data from a demographic surveillance system in rural KwaZulu-Natal (2000–2014), we estimate differences between male and female adult life expectancy by HIV status. The contribution of causes of death to these life expectancy differences are computed with demographic decomposition techniques. Cause of death information comes from verbal autopsy interviews that are interpreted with the InSilicoVA tool.

**Results:**

Adult women lived an average of 10.4 years (95% confidence Interval 9.0–11.6) longer than men. Sex differences in adult life expectancy were even larger when disaggregated by HIV status: 13.1 (95% confidence interval 10.7–15.3) and 11.2 (95% confidence interval 7.5–14.8) years among known HIV negatives and positives, respectively. Elevated male mortality from pulmonary tuberculosis (TB) and external injuries were responsible for 43% and 31% of the sex difference in life expectancy among the HIV negative population, and 81% and 16% of the difference among people living with HIV.

**Conclusions:**

The sex differences in adult life expectancy in rural KwaZulu-Natal are exceptionally large, atypical for an African population, and largely driven by high male mortality from pulmonary TB and injuries. This is the case for both HIV positive and HIV negative men and women, signalling a need to improve the engagement of men with health services, irrespective of their HIV status.

## Introduction

Women live, on average, five years longer than men. This difference varies across countries and across time, with larger differences typically found in populations with lower mortality [1]. In high mortality populations, sex differences are often attenuated by relatively high maternal mortality rates and, in the case of some African populations of the late 1990s and early 2000s, relatively high HIV prevalence and HIV/AIDS-associated mortality among women [1, 2]. As life expectancy (LE) increases, the relative importance of mortality from maternal and infectious causes declines and mortality differences between men and women tend to increase [3–6].

**Fig 1:**
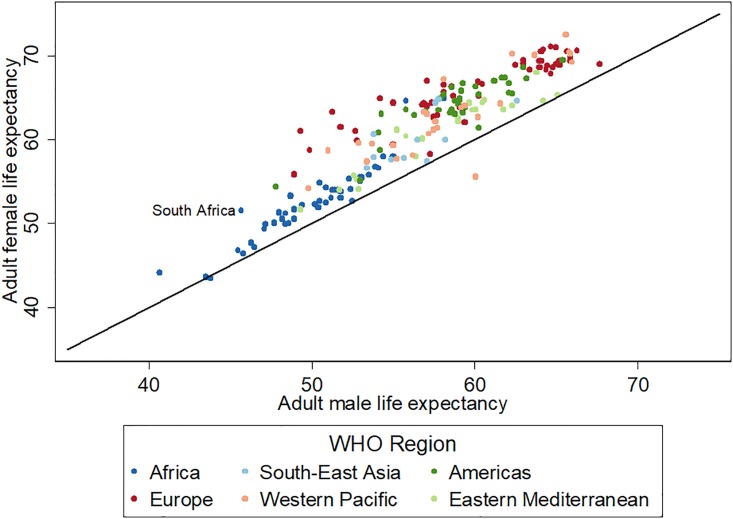
Adult life expectancy by sex and WHO region, 2013. Source: WHO Global Health Observatory Data, http://www.who.int/gho/en/.

The correlation between mortality levels and the magnitudes of the sex differences is illustrated in a scatterplot of adult male and female LE from 192 countries (Fig 1). In 2013, Europe had an adult LE at age 15 (for both sexes combined) of 62.3 years and a sex difference of 7.1 years, whereas the African region had an adult LE of 51.1 years and a sex difference of 2.5 years [1]. With a gap of 5.9 years, the sex disparity in South Africa approaches the European average, but the absolute adult LE levels are more alike other African countries.

The district of uMkhanyakude in the South African province of KwaZulu-Natal has an even larger adult LE difference than that observed at the national level [2]. In a recent paper, Bor et al. demonstrated that sex differences in uMkhanyakude decreased in the years that the impact of HIV on adult mortality started to materialize. After antiretroviral therapy (ART) became available in 2004, sex differences in adult LE began to increase again, reaching 8.6 years in 2011. This fluctuation in the LE difference between men and women is attributed in part to the relatively high HIV prevalence among women. As a result, women lost more life years as the HIV epidemic unfolded and, consequentially, had more life-years to gain from the expansion of treatment. This phenomenon is reinforced by two other factors, namely women’s younger ages at infection [7, 8] and women’s lower mortality from causes unrelated to HIV. In the absence of treatment, a typical female HIV/AIDS-associated death will therefore incur a larger loss in LE than a male HIV/AIDS death. Conversely, preventing a female HIV/AIDS death will entail a larger gain in life-years. In addition to these demographic factors, Bor et al. noted that the faster uptake of life-saving HIV treatment among women led to more rapid declines in HIV/AIDS-related mortality in women than in men [2, 9].

In this study, we illustrate that the sex disparity in adult LE is equally large in the HIV negative population as among people living with HIV (PLHIV). Further, we use verbal autopsy data on causes of death to illustrate that pulmonary TB and injuries are the two factors driving the large sex differences in adult mortality.

## Materials and methods

### Data and study population

Data for this study come from a demographic surveillance system (DSS) in the uMkhanyakude district of northern KwaZulu-Natal, which is also known as the Population Intervention Platform (formerly ACDIS). The study area covers 438 km^2^ of mostly rural land and has a population characterised by high HIV prevalence [10], a young age structure, and high levels of circulatory migration [11]. KwaZulu-Natal also has one of the most severe TB epidemics in the world with case notification rates that exceeded 1066 per 100,000 in 2006, and a high prevalence of multiple drug resistant TB [12]. The public sector ART programme in the study area enrolled its first patients in August 2004. By mid-2011 an estimated 37% of PLHIV in the study population were on ART [13, 14].

Since 2000, vital events data have been collected on all members of a household, whether they ordinarily reside in the study site or not. We do not, however, count exposure time lived outside the study area. Similarly, we exclude time observed before age 15, and—to minimize possible bias from age over-reporting—after age 100. The sensitivity of our results to these inclusion criteria are discussed in the supporting information (S2 File). The data extraction from the DSS database was done in August 2015, and observations were administratively or right censored at the end of 2014; the last calendar year with near complete data. The data for 2014 are not complete because some households may be skipped whenever no eligible member is present when the enumerators visit. The data for these households are updated during the next census round and not included here.

HIV status information comes from HIV seroprevalence surveys that have been conducted annually since 2003. Initially only men and women of reproductive age were invited to participate in the serosurveys; in 2007, the age-eligibility criterion was expanded to all adults [11]. The year 2007 is therefore also used as the starting point for mortality estimates by HIV status. The HIV serosurvey information is complemented by record linkage with HIV treatment and care data collected at primary health care clinics serving the DSS residents. HIV status information in the dataset is classified as unknown prior to an individual’s first HIV test. In the time following an HIV negative result, an individual’s status is classified as negative for five years, at which point their status is changed to unknown. An individual is considered HIV positive following their first positive test and that information is not censored or stale-dated. The person-time for which HIV status is known to the study gradually increased, reaching 57% in 2014. Elsewhere, we provide more detail on the HIV status information in the dataset, and justify the cut-off for the exposure time following an HIV negative test [15]. HIV serosurvey participation dynamics are discussed in Larmarange et al [16].

Verbal autopsy (VA) interviews are routinely conducted with a close caregiver for all deceased individuals. The VA questionnaire evolved over the years, and now conforms to the INDEPTH/WHO standard [17]. VA surveys were incomplete for 395 deaths (4%) and were not used in the cause of death determination.

Ethical approval for this study was obtained from the Biomedical Research Ethics Committee of the University of KwaZulu-Natal and the Observational Research Ethics Committee of the London School of Hygiene & Tropical Medicine. Household representatives gave verbal informed consent for the demographic surveillance, and individual written consent was obtained for HIV testing and the retrieval of information from medical records.

### Statistical analysis

Sex differences in adult LE are reported for the entire population since 2000 and by known HIV status since 2007. Adult LE is defined as the number of additional years that a 15-year-old can expect to live under the mortality conditions that prevail in a calendar year or period (see S1 File). Annual estimates of adult LE are computed with continuous-time survival analysis techniques as the area under Kaplan Meier survival curve. In the epidemiological literature, this quantity is also referred to as the (restricted) mean survival time [18]. Percentile-based confidence bounds for the sex differences of these estimates are obtained via bootstrapping with 1,000 replications.

The inquiry into the sex differences in adult mortality is complemented with an age-cause decomposition of adult LE differences between men and women in 2010–2014 using a method first described by Arriaga [19]. We use a five calendar year time span to accumulate sufficient data to stratify by HIV status, and decompose the total sex difference into contributions resulting from mortality differences in each five-year age group. Using cause of death (CoD) information and making the assumption that the distribution of cause-specific deaths is constant in each five-year age interval, we then estimate the cause contributions to the sex differences in adult LE for each age group by multiplying the cause-specific mortality fractions (CSMFs) with the age group contribution to the sex difference in LE [20]. The age-cause decomposition is done separately for each HIV status category. It is also worth pointing out that our estimates rest on the independent competing risks assumption, which presumes that the removal of one cause will leave the risk of dying from all other causes unchanged [21].

To assign causes of death, we use the InSilicoVA tool [22, 23]. InSilicoVA is a VA interpretation tool that uses the signs and symptoms reported by the deceased’s caretaker during the verbal autopsy interview to assign the presumed cause of death. InSilicoVA uses a Bayesian model to estimate both CSMFs for pre-specified sub-populations and cause-specific probabilities at the individual level. Due to the relatively low numbers in some five-year age groups, we estimate CSMFs for the sub-populations defined by two broad age groups (below 60 and 60 or older), sex, and HIV status. We then obtain empirical CSMFs for each five-year age group by aggregating individual probabilities. The CoD classification scheme used for reporting the results is based on the Global Burden of Disease studies: HIV/AIDS-related deaths, pulmonary tuberculosis (TB), other communicable diseases, malignant neoplasms, cardiovascular disease, other non-communicable diseases, external causes/injuries, and maternal mortality. Even though, we separately report on TB and HIV/AIDS-associated mortality in our results, it is well-known that both causes are very hard to distinguish for HIV positives because of the similarity of symptoms and high co-morbidity [24, 25]. The mapping of the CoD groups onto the ICD10 codes is provided in Table A in S3 File. In other appendices we report CSMFs (S4 File), analyses for individuals whose HIV status is unknown to the study (S5 File), and a set of results where the CoD assignment has been done with the R-version of InterVA (S6 File) [26, 27].

## Results

Between 2000 and 2014, 95,899 adults (52,751 women) lived in the DSS area and jointly contributed 571,163 person-years and 10,680 deaths to the dataset. In the period during which HIV testing was available for all adults (2007–2014), HIV negative adults contributed 34% of the person-years lived in adulthood and PLHIV contributed 17%.

In 2010–2014, there were 2,760 deaths resulting in a crude adult death rate of 12.7 deaths per 1,000 person-years (95% confidence interval: 12.0, 13.3) among women, and 16.3 deaths per 1,000 person-years (95% confidence interval: 15.5, 17.2) among men (Table 1). Crude mortality rates among HIV negative individuals were similar to those of the entire population, with an all-cause female mortality rate of 12.7 deaths per 1,000 person-years (95% confidence interval: 11.7, 13.8) and a male mortality rate of 16.4 deaths per 1,000 person-years (95% confidence interval: 14.9, 18.0). Among PLHIV, sex differences in mortality were more explicit with an all-cause female mortality rate of 18.9 deaths per 1,000 person-years (95% confidence interval: 17.3, 20.6) and a male mortality rate of 46.2 deaths per 1,000 person-years (95% confidence interval: 42.0, 50.8).

**Table 1:** Summary statistics by gender and HIV status, 2010–2014.

HIV status^1^	Sex	Number of individuals^2^	Person-years of follow up	Number of deaths (%)	Crude death rate per 1,000 PY (95% CI)^3^
**Negative**	*Female*	13,519	41,282.3	524 (3.9)	12.7 (11.7, 13.8)
	*Male*	10,042	25,983.9	425 (4.2)	16.4 (14.9, 18.0)
	*Total*	23,561	67,266.3	949 (4.0)	14.1 (13.2, 15.0)
**Positive**	*Female*	9,070	27,445.3	518 (5.7)	18.9 (17.3, 20.6)
	*Male*	3389	9,223.4	426 (12.6)	46.2 (42.0, 50.8)
	*Total*	12,459	36,668.7	944 (7.6)	25.7 (24.2, 27.4)
**Unknown**	*Female*	21,351	44,432.9	391 (1.8)	8.8 (8.0, 9.7)
	*Male*	20,507	46,104.3	476 (2.3)	10.3 (9.4, 11.3)
	*Total*	41,858	90,537.2	867 (2.1)	9.6 (9.0, 10.2)
**All**	*Female*	33,804	113,160.6	1,433 (4.2)	12.7 (12.0, 13.3)
	*Male*	26,380	81,311.6	1,327 (5.0)	16.3 (15.5, 17.2)
	*Total*	60,184	194,472.2	2,760 (4.6)	14.2 (13.7, 14.7)

Notes:

^1^ We report the HIV status information as it is known to the study and may not be the same as men and women’s knowledge of their own HIV status. Unknown HIV status includes all the persons-years of exposure before the start of the HIV surveillance as well as individual time prior to the first HIV test, and exposure time more than five years after the last HIV negative test.

^2^ The sum of the number of individuals within each HIV status group is greater than the total number of individuals in the study because an individual’s HIV status can change during the study period. Similarly, individuals can contribute observation time to more than one HIV status group.

^3^ The crude death rate of HIV negatives is about the same as for all adults combined, a result that is the consequence of the relatively old age structure of the HIV negative population [15].

**Fig 2:**
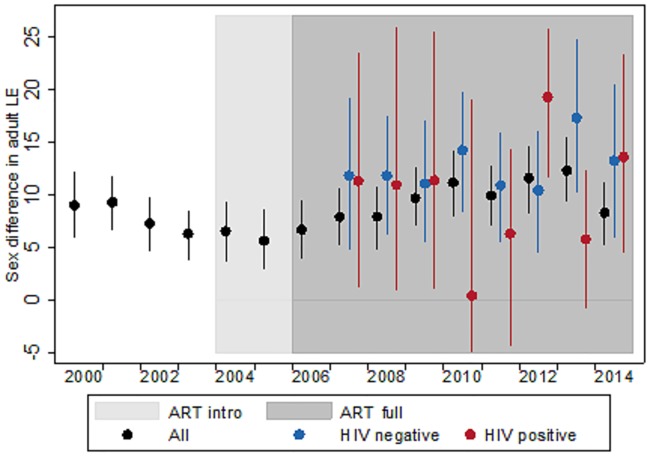
Trends of the female advantage in adult life expectancy (overall and by HIV status), 2000–2014. Notes: ^1^ The difference is the average number of extra years that adult women are expected to live compared to adult men in uMkhanyakude. ^2^ The confidence interval for the HIV positive population in 2010 has been truncated and should extend from -21.7 to 19.0.

Fig 2 illustrates trends in the sex differences in adult LE. The sex difference for the population as a whole first contracted, and again increased following the introduction of ART in 2004. We estimated that women lived 9.0 years (95% confidence interval: 5.9, 12.2) longer than men in 2000, 5.7 years (95% confidence interval: 3.0, 8.6) longer in 2005, and 12.3 years (95% confidence interval: 9.3, 15.5) longer in 2013. The sex difference appears to have declined again in 2014, but we note that the estimates for 2014 are not based on data for a full calendar year.

The average sex difference for the period 2007–2014 was 9.6 years (95% confidence interval: 8.6, 10.6) for the entire population, and is even larger for men and women with a known HIV status. This is particularly the case for HIV negatives (13.1 years, 95% confidence interval: 10.7, 15.3); the sex difference among PLHIV is also larger than the population average, but is estimated with greater uncertainty (11.2 years, 95% confidence interval: 7.5, 14.8). Detailed estimates are reported in Table A in S1 File.

**Fig 3:**
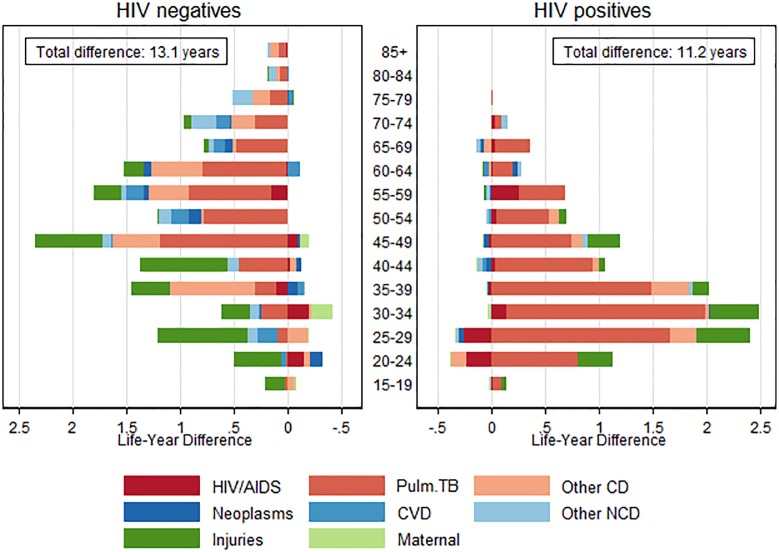
Age-cause decomposition of the female advantage in adult life expectancy of the known HIV negative and HIV positive populations, 2010–2014. Note: Positive values in these plots indicate that the cause-specific mortality rates in a particular age group are higher for men than for women and thus increase the female advantage in adult LE; negative values suppress the female LE advantage. Note that the axis for HIV negatives has been reversed.

Fig 3 summarizes the age-cause decomposition of the sex differences in adult LE by HIV status. Results for men and women whose HIV status is unknown are reported in S5 File. The decomposition is done for the 2010–2014 period because the numbers are too small to facilitate an analysis by calendar year. The total LE difference in this period was 10.4 years for the population as a whole, 13.1 years among HIV negatives, and 11.2 years among PLHIV. Each horizontal bar in Fig 3 represents the contribution of age-specific mortality differences to the total female mortality advantage. These contributions are further decomposed by broad CoD group. Negative values in these plots indicate that the mortality rates from a particular cause in a specific age group are higher for women than for men, and thus suppressed the sex difference in adult LE. Their contributions are small. Table 2 aggregates the CoD contributions across all ages groups and gives the total contribution of each cause to the LE difference between men and women.

**Table 2:** Contributions of causes of death to the total sex difference in adult life expectancy, by HIV status (2010–2014).

Cause of death	HIV negative		HIV positive	
	Sex life-year difference	%*	Sex life-year difference	%*
HIV/AIDS related	-0.2	-	0.0	0.4
Pulmonary tuberculosis	5.6	40.9	9.0	78.6
Other communicable diseases	2.3	16.8	0.6	5.2
Malignant neoplasms	-0.0	-	-0.1	-
Cardiovascular disease	0.6	4.4	-0.1	-
Other non-communicable diseases	1.1	8.0	-0.0	-
Maternal causes	-0.3	-	-0.1	-
Injuries	4.1	29.9	1.8	15.7
Total	13.1	100	11.2	100

Notes:

* Percent of the sum of positive differences in adult.

Two thirds of the female LE advantage among HIV negative adults was due to mortality differences above age 45, and associated with excess male deaths from communicable diseases. Almost half of the total LE gap (5.6 years) was attributed to higher pulmonary TB mortality among men, while other communicable diseases were responsible for an additional female LE advantage of 2.3 years. Elevated rates of external injuries among men accounted for 4.1 years of the total sex difference in adult LE. Most of the sex difference in external injuries (2.9 years) was accrued below age 50. Higher rates of cardiovascular disease and other non-communicable disease mortality among men contributed another 1.7 years to the total sex gap.

In contrast to the HIV negative population, most of the female LE advantage among PLHIV was due to higher mortality among men under 45 years, especially higher male mortality from pulmonary TB. Pulmonary TB alone accounted for an LE difference of 9.0 years. Higher HIV/AIDS-related mortality among younger women slightly offset the high pulmonary TB mortality rates among men in some of the younger age groups, but when summed across all ages, deaths explicitly attributed to HIV had a negligible effect on the sex disparity in adult LE.

After pulmonary TB, external injuries were the second largest contributor to women’s mortality advantage among PLHIV, contributing 1.8 years to the LE differential. Most of the excess mortality from external causes occurred before age 45. High male mortality from other communicable diseases was responsible for a further 0.6 years of the sex differential.

The decomposition using InterVA-4’s CoD attribution also indicated that pulmonary TB and injuries were the primary drivers of the sex differential in the HIV negative population and thus supports the findings based on InSilicoVA. Both VA interpretation tools attributed similar fractions of the difference to injury deaths (S6 File). Small differences are, however, worth mentioning. In the HIV negative population, InterVA-4 attributed 1.2 fewer years of the sex difference to pulmonary TB and 1.9 fewer years to other communicable diseases. Instead, InterVA-4 attributed more of the sex differences in the life-years lived in adulthood to non-communicable disease mortality (2.6 years in total). Among PLHIV, InterVA again attributed a smaller fraction of the difference to pulmonary TB (1.6 years), although it still indicated that pulmonary TB was the primary driver of the sex difference. InterVA-4 was more likely to attribute the sex difference in mortality at younger ages to differences in HIV/AIDS-related mortality, and more of the difference at higher ages to non-communicable diseases.

## Discussion

Large sex differentials in LE are typical of high-income countries with low mortality levels. In these settings, the female mortality disadvantage associated with elevated maternal mortality has dissipated and excess male mortality results from cardiovascular disease, neoplasms, and injuries [28–31]. With an adult LE of 51.8 years for women and 41.4 years for men in 2010–2014, this population in KwaZulu-Natal does not qualify as a low mortality population, yet it has a sex gap in excess of 10 years, which is more than four times the WHO’s 2013 estimate of 2.5 years for the African region as a whole [1]. Aside from the sheer size of the difference with the regional average, there are two other reasons that render these results remarkable.

First, HIV prevalence in this study population is very high and that usually suppresses sex differences in LE. This is because (i) HIV prevalence is often higher among women than among men [32]; (ii) women are generally infected at younger ages than men and thus die younger; and (iii) women have, on average, fewer competing mortality risks in adulthood. An HIV/AIDS-associated female death thus incurs a greater loss in life-years than a male HIV/AIDS death. The consequence is a reduction in the LE difference between men and women as the mortality impact of HIV increases, which reverses when ART is rolled out and the impact of HIV on mortality dissipates [2, 33]. The expansion of gender differences in recent years is reinforced by women’s better engagement with HIV care and treatment services, which has since ART resulted in larger mortality reductions among female HIV positives than among HIV positive men [2, 9]. We return to this matter below.

Second, the sizable sex difference in adult LE persists in estimates that are disaggregated by HIV status. More specifically, it is the large disparity in the LE of HIV negative men and women that stands out, and this phenomenon results from disproportionally high male mortality. In 2013, HIV negative women in the study population lived an average of 62.6 (95% confidence interval: 59·5–65·5) years past their fifteenth birthday, which is close to the adult LE of 63.1 years for women in upper-middle income countries. In contrast, HIV negative men in KwaZulu-Natal had an adult LE of 46.1 years (95% confidence interval: 41.7–50.7), while the estimate for men in upper-middle income countries was 59.1 years [1].

Our inquiry into the causes that contribute to the relatively high mortality in HIV negative men points at pulmonary TB and injuries. Depending on the VA interpretation tool that is used, pulmonary TB alone is responsible for a sex difference in adult LE of 4.4 (InterVA) or 5.6 (InSilicoVA) years. The contribution of pulmonary TB to the gender disparity in adult mortality is thus extremely large, which lends support to studies in African populations that have documented the relatively high prevalence of TB among men, as well as the failure to reach or engage men with preventative, diagnostic and treatment programs [34–37].

Excess male mortality from injuries account for a LE difference of around 4 years in HIV negatives (irrespective of the VA interpretation tool that is used), and are in agreement with studies that have documented the high the burden of injury mortality, interpersonal violence in particular, in South African men [38, 39].

Gender differences in the mortality of PLHIV are also large, and result from sex disparities in the engagement with HIV services (women have higher HIV diagnosis and ART coverage rates [40–42], and lower attrition and mortality rates after initiating ART [42–46]), which ultimately leads to different mortality rates from HIV/AIDS-associated causes, including TB. On the basis of the VA interviews, we estimate that about 81% of the sex difference in the adult LE among PLHIV is attributable to pulmonary TB. We reiterate that pulmonary TB is difficult to differentiate from HIV among PLHIV, but the relatively high mortality from pulmonary TB among HIV negative men suggests that some of the disparity in the mortality rates of PLHIV arises from sex differences in co-infection rates with TB.

Interpretation of our findings is subject to the limitations of verbal autopsies for classifying causes of death [47, 48]. The differences between the two VA interpretation tools used in this study are relatively minor, but it is worth noting that the InterVA-4 model ascribes somewhat lower importance to pulmonary TB for the large sex differences in adult mortality. Among PLHIV, this is compensated with a larger differential explicitly attributed to HIV; among HIV negative men and women this is compensated with a larger fraction of male deaths ascribed to neoplasms and cardiovascular disease.

In conclusion, these results testify to the disproportionately high burden of mortality from TB and external injuries among men in in KwaZulu-Natal, and signal the need to improve efforts to target men with preventative, diagnostic and curative health services.

## Supporting information

S1 File Estimates of the sex differences in adult LE by calendar year and HIV status. (DOCX)

S2 File Sensitivity of the estimates to the exclusion of non-residents. (DOCX)

S3 File Cause of death classification scheme. (DOCX)

S4 File Cause-specific mortality fractions by HIV status and sex. (DOCX)

S5 File Causes of death among men and women whose HIV status is unknown. (DOCX)

S6 File Analyses with the InterVA-4 model for cause-of-death attribution. (DOCX)
