# Supplementary material for: Tuberculosis mortality and the male survival deficit in rural South Africa: An observational community cohort study
Source: PLoS One. 2017 Oct 10;12(10):e0185692. doi: 10.1371/journal.pone.0185692 (PMC5634548; doi:10.1371/journal.pone.0185692)
Supplement: S1 File — (DOCX) [file pone.0185692.s001.docx]

**S1 File. Estimates of the sex differences in adult LE by calendar year and HIV status**

Table A provides annual estimates of the sex differences in adult life expectancy in uMkhanyakude. These are estimated using continuous-time survival analysis techniques as the area under Kaplan Meier survival curve. Percentile-based confidence intervals are obtained via bootstrapping with 1,000 replications. Estimates by HIV status are only provided from 2007 onwards, the point at which the age eligibility criteria were expanded to all adults. The estimates reported in Table A are the basis for Fig 3 in the main text.

**Table A. Sex difference in adult life expectancy by HIV status and calendar year**

| Year | HIV Negative | | HIV Positive | | Unknown | | All | | |  |
| --- | --- | --- | --- | --- | --- | --- | --- | --- | --- | --- |
|  | **Estimate^1^** | **95% CI^2^** | **Estimate** | **95% CI** | **Estimate** | **95% CI** | | **Estimate** | **95% CI** | |
| 2000 | / |  | / |  | / |  | | 9.0 | 5.9-12.2 | |
| 2001 | / |  | / |  | / |  | | 9.3 | 6.6-11.8 | |
| 2002 | / |  | / |  | / |  | | 7.2 | 4.7-9.8 | |
| 2003 | / |  | / |  | / |  | | 6.3 | 3.7-8.5 | |
| 2004 | / |  | / |  | / |  | | 6.5 | 3.6-9.3 | |
| 2005 | / |  | / |  | / |  | | 5.7 | 3.0-8.6 | |
| 2006 | / |  | / |  | / |  | | 6.7 | 3.9-9.4 | |
| 2007 | 11.8 | 4.6-19.0 | 11.3 | 1.2-23.6 | 10.0 | 6.4-13.7 | | 7.9 | 5.2-10.6 | |
| 2008 | 15.3 | 9.7-20.7 | 10.9 | 1.0-25.7 | 6.3 | 1.3-10.9 | | 7.9 | 4.7-10.7 | |
| 2009 | 13.7 | 8.1-19.1 | 11.4 | 1.4-26.3 | 10.5 | 6.2-15.0 | | 9.7 | 7.0-12.6 | |
| 2010 | 15.0 | 9.5-20.2 | 0.4 | -21.5-18.5 | 8.1 | 3.4-12.9 | | 11.1 | 8.0-14.1 | |
| 2011 | 12.5 | 6.8-17.9 | 6.3 | -3.6-14.1 | 11.3 | 6.0-16.4 | | 9.9 | 7.1-12.7 | |
| 2012 | 13.2 | 7.6-18.1 | 19.3 | 10.5-26.2 | 5.9 | 0.2-10.9 | | 11.6 | 8.2-15.5 | |
| 2013 | 16.5 | 10.9-21.8 | 5.8 | -0.8-12.4 | 12.9 | 7.7-18.1 | | 12.3 | 9.3-15.5 | |
| 2014 | 12.6 | 6.7-17.9 | 13.6 | 4.4-23.3 | 4.8 | -0.5-10.3 | | 8.3 | 5.2-11.2 | |

When calculating adult LE estimates we discarded person-years lived above age 100 due to potential bias from age over-reporting. The precise interpretation of our LE measure is therefore expected number of years lived between ages 15 and 100 given the age-period mortality rates. In effect, we truncated the lifelines of 31 individuals and dropped 82.4 person-years of observation time. These individuals were mostly female (25/31) and were either HIV negative (18/31) or had an unknown HIV status (13/31). Excluding the person-years lived above age 100 reduced the adult LE estimate of all women by 0.5 years, of HIV negative women by 0.9 years, and of women with an unknown status by 0.3 years. Changes among PLHIV and among men were negligible. Given that the inclusion criteria affected women more than men, the LE difference would have been larger with the inclusion of the exposure time of centenarians: by 0.4 years for the population as a whole, and by 0.8 years for HIV negatives.
