# Supplementary material for: Tuberculosis mortality and the male survival deficit in rural South Africa: An observational community cohort study
Source: PLoS One. 2017 Oct 10;12(10):e0185692. doi: 10.1371/journal.pone.0185692 (PMC5634548; doi:10.1371/journal.pone.0185692)
Supplement: S2 File — (DOCX) [file pone.0185692.s002.docx]

**S2 File. Sensitivity of the estimates to the exclusion of non-residents**

We exclude the exposure time of non-resident household members because they have lower participation rates in the HIV serosurveys and because their verbal autopsy information may be less accurate since the respondent may not have been co-residing with the deceased during the final illness episode. Additionally, non-residents may have been exposed to a different set of mortality risks than migrants [1]. Fig A illustrates the trends in sex differences in adult LE using only resident episodes and both resident and non-resident episodes.

The exclusion of non-residents affected men more than women; men’s LE mostly increased when including non-resident household members, women’s LE fluctuated in either direction but usually by less than a year. As a result, the sex difference in LE decreased with a maximum of 3.4 years when including non-residents in the analyses.

**Fig A. Annual estimates of the sex difference in adult LE among residents alone and among both residents and non-residents.**

**Reference**

1. Welaga P, Hosegood V, Weiner R, Hill C, Herbst K, Newell ML. Coming home to die? The association between migration and mortality in rural South Africa. BMC Pub Health. 2009;9:193. doi: 10.1186/1471-2458-9-193. PubMed PMID: 19538717; PubMed Central PMCID: PMCPMC2706824.
