# Supplementary material for: Tuberculosis mortality and the male survival deficit in rural South Africa: An observational community cohort study
Source: PLoS One. 2017 Oct 10;12(10):e0185692. doi: 10.1371/journal.pone.0185692 (PMC5634548; doi:10.1371/journal.pone.0185692)
Supplement: S3 File — (DOCX) [file pone.0185692.s003.docx]

**S3 File. Cause of death classification scheme**

Table A maps the cause of death groups used in the manuscript against the codes from the International Statistical Classification of Diseases and Related Health Problems (ICD-10). More information on the ICD-10 is available at <http://www.who.int/classifications/icd/en/>.

# Table A: Causes of death used in the manuscript their associated ICD-10 code

| Cause of Death (CoD) groups in manuscript | ICD-10 code |
| --- | --- |
| HIV/AIDS related | B20-B24 |
| Pulmonary tuberculosis | A15-A16 |
| Other communicable diseases & nutritional conditions | A00-A09; A17-A99; B00-B19; B25-B99; D50-D64; E40-E46; G00-G05; J00-J22 |
| Maternal disorders | O00-O08; O10-O16; O20-O99 |
| Malignant neoplasms | C00—C26; C30-C58; C60-D48 |
| Cardiovascular diseases | D57; I00-I15; I20-I52; I60- I99 |
| Other non-communicable diseases | D55-D89; E00-E07; E10-E35; E50-E90; F00-F99; G06—G37; G40-G41; G50-G99; H00-H95; J30-J99; K00-K31; K35-K38; K40-K93; L00-L99; M00-M99; N00-N99; R00-R94 |
| External injuries | S00-T99; V01-V99; W00-W99; X00-X99; Y00-Y98 |
| Indeterminate | R95-R99 |
