# Supplementary material for: Tuberculosis mortality and the male survival deficit in rural South Africa: An observational community cohort study
Source: PLoS One. 2017 Oct 10;12(10):e0185692. doi: 10.1371/journal.pone.0185692 (PMC5634548; doi:10.1371/journal.pone.0185692)

**S4 File. Cause-specific mortality fractions by HIV status and sex**

Cause-specific mortality fractions are the proportions or percentages of deaths attributable to each broad cause-group and sum up to 100. In Fig A below, the cause of death attribution is based on VA interviews interpreted with the InSilicoVA model. In the HIV negative population, just over half of the deaths among both women and men were attributed to communicable causes. Despite that similarity, pulmonary TB deaths were three times more common among men (29%) than among women (10%). Under 3% of the mortality among HIV negatives is attributed to HIV/AIDS, which could be due to both misclassification of HIV status or misclassification of the cause of death. Error in the HIV status could arise from a false negative HIV test result or from HIV serconversion (and death) within five years of the last HIV negative test.

Noncommunicable diseases were a more common CoD for HIV negative women than men. This was particularly the case for cardiovascular diseases, which accounted for 20 versus 10% of the deaths among men and women, respectively, and deaths to neoplasms (5 versus 3%). In contrast, a higher proportion of men died from external causes (16 versus 6%). Maternal causes were ascribed to 1% of the deaths among HIV negative women.

Among PLHIV, similar proportions of female and male deaths (87% and 88%, respectively) were attributed to communicable causes, but with a higher proportion of female deaths explicitly attributed to HIV/AIDS (45% versus 21%). In return, a higher proportion of male deaths were attributed to pulmonary TB (57% versus 30%). Deaths from neoplasms, cardiovascular disease, and other non-communicable diseases caused 9% of all female deaths and 3% of all male deaths. As was the case for the HIV negative population, deaths from external causes were twice as common among men compared to women (9 versus 4%), and fewer than 1% of female deaths were attributed to maternal causes.

**Fig A. Cause-specific mortality fractions for adult deaths, by HIV status and sex (InSilicoVA, 2010-2014)**

1. **HIV negative (b) HIV positive**

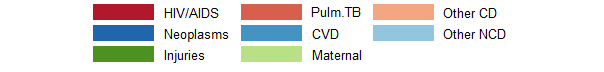

Supplement: S4 File — (DOCX) [file pone.0185692.s004.docx]
