# Supplementary material for: Tuberculosis mortality and the male survival deficit in rural South Africa: An observational community cohort study
Source: PLoS One. 2017 Oct 10;12(10):e0185692. doi: 10.1371/journal.pone.0185692 (PMC5634548; doi:10.1371/journal.pone.0185692)

**S5 File. Causes of death among men and women whose HIV status is unknown**

**1. Cause-specific mortality fractions**

Fig A gives the cause-specific mortality fractions for men and women whose HIV status is not known to the study. There was only a small sex difference in the proportion of deaths attributed to all communicable diseases, but some differences are noticeable in the more specific cause groups: HIV/AIDS was the presumed cause for 17% of female deaths and 9% of male deaths, while pulmonary TB accounted for 33% of male deaths and 15% of female deaths. Noncommunicable diseases were responsible for 29% of deaths among women, and 16% of deaths among men. External causes accounted for 23% of deaths among men, and for 5% of deaths among women. Around 1% of the deaths to women were attributed to maternal causes.

**Fig A. Cause-specific mortality fractions among adults whose HIV status was unknown to the study, by sex (InSilicoVA, 2010-2014)**


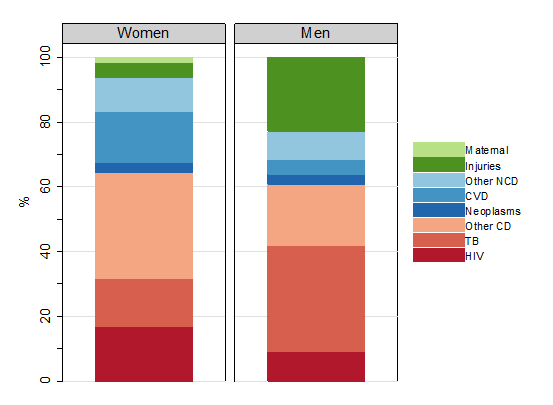

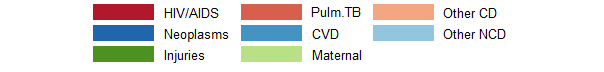


**2. Age-cause decomposition of sex differences in adult LE**

In the period 2010-1014, women with an unknown HIV status had an adult LE of 55.3 years, and men had an adult LE of 46.6 years, which amounts to a sex difference of 8.6 years (95% confidence interval: 6.5,11.0). Half of the LE difference had been accrued by the age of 49, which is comparable to the age profile of the sex differences in the population as a whole.

Pulmonary TB and injuries respectively contributed 4.9 years (57%) and 3.6 years (41%) to the sex difference (Fig B). Excess female mortality explicitly attributed to HIV/AIDS suppressed the LE gap by 1.1 years.

**Fig B. Age-cause decomposition of the sex difference in adult LE in the population whose HIV status is unknown (InSilicoVA, 2010-2014)**

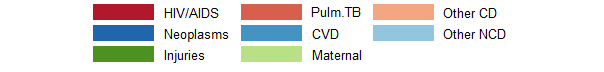

Supplement: S5 File — (DOCX) [file pone.0185692.s005.docx]
