# Supplementary material for: Tuberculosis mortality and the male survival deficit in rural South Africa: An observational community cohort study
Source: PLoS One. 2017 Oct 10;12(10):e0185692. doi: 10.1371/journal.pone.0185692 (PMC5634548; doi:10.1371/journal.pone.0185692)
Supplement: S6 File — (DOCX) [file pone.0185692.s006.docx]

**S6 File. Analyses with the InterVA-4 model for Cause-of-Death (CoD) attribution**

All analyses were repeated with the InterVA-4 model to assign causes of death [1]. We used the R version (v1.5) of the model with the replicate option set to false, which fixes known bugs from InterVA v.4.02 [2]. InterVA generates one to three probable CoDs with their corresponding likelihoods (not necessarily summing to 100%) or an indeterminate CoD with 100% likelihood. If the likelihood does not sum to 100%, the residual fraction is assigned an indeterminate status. Likelihoods were summed by sex, age group, HIV status, and CoD group to estimate cause-specific mortality fractions. As with the analyses based on InSilicoVA, deaths with incomplete VAs were excluded.

The model was run with malaria prevalence set to low and HIV prevalence set to high for the known HIV positive population, and with both malaria and HIV prevalence set to low for the known HIV negative population. CoD attribution was done separately by HIV status to minimize misclassification of HIV-associated deaths. InterVA uses the same CoD classification scheme as InSilicoVA, but does also classify a portion of the deaths as indeterminate. In contrast, InSilicoVA attributes deaths with weakly specified causes to the residual categories of other communicable and other non-communicable diseases.

**Fig A. Cause-specific mortality fractions for adult deaths using InterVA-4 to assign cause of death, by sex and HIV status (2010-2014)**

1. **HIV negative (b) HIV positive**

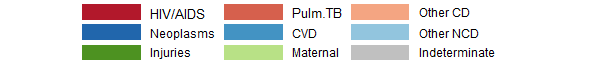


Fig A summarizes the cause-specific mortality fractions by sex and HIV status. In the HIV negative population, non-communicable diseases were responsible for the majority (74%) of female deaths and just over half (54%) of male deaths, and communicable diseases caused more male deaths than female deaths (30% and 17%, respectively). Cardiovascular disease was the primary killer of women (30%); and pulmonary TB was the leading killer among men (22%). Injury-related deaths were also common among men (15%), but not as frequent among women (6%). InterVA-4 was unable to determine the CoD for 3% of women and 1% of men.

Among PLHIV, approximately three quarters of all deaths were caused by HIV/AIDS or TB (73% of female deaths and 78% of male deaths), with a higher proportion of women dying from HIV/AIDS than men, and a higher proportion of men dying from TB. Non-communicable diseases were responsible for 11% of male deaths and 13% of female deaths. External causes accounted for 8% and 3% of male and female deaths, respectively. Among PLHIV, the cause of death could not be determined for 5% of female deaths and under 1% of male deaths.

The age-cause decomposition of the sex differences in adult LE by HIV status are shown in Fig B. Table A aggregates the contributions for each cause over age. Among HIV negatives, the female mortality advantage mostly resulted from lower mortality rates due to pulmonary TB, injuries, and non-communicable diseases. Differences in injury-related mortality contributed 4.0 years to the female LE advantage, and this was mostly accrued among individuals under 45 years of age. Excess male mortality from pulmonary TB and non-communicable diseases were responsible for a female advantage of 4.4 and 4.3 years, respectively, and this was mostly accrued among individuals above age 45.

Among PLHIV, pulmonary TB was the most common cause for men’s excess mortality, accounting for 7.4 years of the 11.2 year gap in adult LE. The second most important factor was HIV/AIDS, which reduced men’s adult LE by 1.9 years compared to women. Excess male mortality from injuries accounted for an additional 1.7 years of the LE difference between men and women.

**Fig B. Age-cause decomposition of the female advantage in adult life expectancy using InterVA-4 to assign cause of death, by HIV status (2010-2014)**

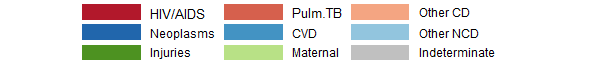


**Table A: Contribution of each cause of death (CoD) to the female advantage in adult life expectancy using InterVA-4 to assign CoD, by HIV status (2010-2014)**

| **Cause of death** | **HIV negative** | | | **HIV positive** | |
| --- | --- | --- | --- | --- | --- |
|  | Sex life-year  difference | % * | Sex life-year  difference | | %* |
| HIV/AIDS related | 0.4 | 3.0 | 1.9 | | 16.3 |
| Pulmonary tuberculosis | 4.4 | 32.6 | 7.4 | | 63.6 |
| Other communicable diseases | 0.4 | 3.0 | -0.0 | | - |
| Malignant neoplasms | 1.3 | 9.6 | 0.4 | | 3.4 |
| Cardiovascular disease | 1.2 | 8.9 | 0.0 | | 0.3 |
| Other non-communicable diseases | 1.8 | 13.3 | 0.2 | | 1.7 |
| Maternal causes | -0.2 | - | -0.1 | | - |
| Injuries | 4.0 | 29.6 | 1.7 | | 14.6 |
| Indeterminate | -0.0 | - | -0.0 | | - |
| Total | 13.1 | 100 | 11.2 | | 100 |

Notes: * Percent of the sum of positive differences in adult.

The two VA interpretation tools lead to similar conclusions regarding the causes of death that are responsible for the female mortality advantage. Among PLHIV, both point at pulmonary TB as the most important cause for the mortality differences, but InterVA explicitly ascribes a non-negligible fraction of the LE disparity to HIV/AIDS. This discrepancy should perhaps not come as a surprise given that both causes are often difficult to separate on the basis of a VA interview [3].

Both models also suggest that pulmonary TB is the most important cause for the gender differences in adult LE. In addition, InSilicoVA attributes a non-negligible share of excess male mortality to unspecified communicable diseases, whereas InterVA ascribes greater importance to neoplasms and cardiovascular diseases as the underlying cause for the sex differences in mortality. Both VA interpretation tools weigh the importance of injuries equally.

**References**

1. Byass P, Chandramohan D, Clark SJ, D'Ambruoso L, Fottrell E, Graham WJ, et al. Strengthening standardised interpretation of verbal autopsy data: the new InterVA-4 tool. Glob Health Action. 2012;5:1-8. doi: 10.3402/gha.v5i0.19281. PubMed PMID: 22944365; PubMed Central PMCID: PMCPMC3433652.

2. Zehang L, McCormick T, Clark S. InterVA4: An R package to analyze verbal autopsy data. In: University of Washington, editor. CSSS Working Paper2014.

3. Glynn JR, Calvert C, Price A, Chihana M, Kachiwanda L, Mboma S, et al. Measuring causes of adult mortality in rural northern Malawi over a decade of change. Glob Health Action. 2014;7. PubMed PMID: WOS:000335337900001.
